# Multi-Contrast MRI Image Synthesis Using Switchable Cycle-Consistent Generative Adversarial Networks

**DOI:** 10.3390/diagnostics12040816

**Published:** 2022-03-26

**Authors:** Huixian Zhang, Hailong Li, Jonathan R. Dillman, Nehal A. Parikh, Lili He

**Affiliations:** 1 Imaging Research Center, Cincinnati Children’s Hospital Medical Center, Cincinnati, OH 45229, USA; huixian.zhang@cchmc.org (H.Z.); hailong.li@cchmc.org (H.L.); jonathan.dillman@cchmc.org (J.R.D.); 2 Department of Radiology, Cincinnati Children’s Hospital Medical Center, Cincinnati, OH 45229, USA; 3 Center for Artificial Intelligence in Imaging Research, Cincinnati Children’s Hospital Medical Center, Cincinnati, OH 45229, USA; 4 Center for Prevention of Neurodevelopmental Disorders, Perinatal Institute, Cincinnati Children’s Hospital Medical Center, Cincinnati, OH 45229, USA; nehal.parikh@cchmc.org; 5 Department of Radiology, University of Cincinnati College of Medicine, Cincinnati, OH 45229, USA; 6 Department of Pediatrics, University of Cincinnati College of Medicine, Cincinnati, OH 45229, USA

**Keywords:** artificial intelligence, CycleGAN, deep learning, MR imaging, pediatric brain, switchable CycleGAN

## Abstract

Multi-contrast MRI images use different echo and repetition times to highlight different tissues. However, not all desired image contrasts may be available due to scan-time limitations, suboptimal signal-to-noise ratio, and/or image artifacts. Deep learning approaches have brought revolutionary advances in medical image synthesis, enabling the generation of unacquired image contrasts (e.g., T1-weighted MRI images) from available image contrasts (e.g., T2-weighted images). Particularly, CycleGAN is an advanced technique for image synthesis using unpaired images. However, it requires two separate image generators, demanding more training resources and computations. Recently, a switchable CycleGAN has been proposed to address this limitation and successfully implemented using CT images. However, it remains unclear if switchable CycleGAN can be applied to cross-contrast MRI synthesis. In addition, whether switchable CycleGAN is able to outperform original CycleGAN on cross-contrast MRI image synthesis is still an open question. In this paper, we developed a switchable CycleGAN model for image synthesis between multi-contrast brain MRI images using a large set of publicly accessible pediatric structural brain MRI images. We conducted extensive experiments to compare switchable CycleGAN with original CycleGAN both quantitatively and qualitatively. Experimental results demonstrate that switchable CycleGAN is able to outperform CycleGAN model on pediatric MRI brain image synthesis.

## 1. Introduction

Magnetic Resonance Imaging (MRI) has been widely utilized in radiology to non-invasively generate images of normal and abnormal anatomy as well as physiological functions of the body [1]. It is a versatile imaging technique that enables the generation of different tissue contrasts depending on the acquisition parameters. For instance, T1-weighted (T1w) MRI increases the signal of fat tissue and decreases the signal of water, while T2-weighted (T2w) MRI increases the signal of water. Taking full consideration of multi-contrast MRI allows for the comprehensive evaluation of scanned organs, potentially improving clinical diagnosis and patient outcomes [2]. However, all the desired contrasts/weightings may not be available due to scan-time limitations, suboptimal signal-to-noise ratio, and/or image artifacts. In addition, an unavailable contrast may also lead to an insufficient data issue for developing robust machine learning and deep learning models [3,4,5,6], which, consequently, may result in poor model performance in the clinical application phase. Thus, there is an urgent research-related and clinical need to synthesize unacquired or corrupted image contrasts using the available image contrast [7].

Deep learning has achieved tremendous progress in cross-modality and the cross-contrast synthesis of medical images. Followed by the early attempts using deep convolutional neural networks (CNNs) [8,9], generative adversarial networks (GANs) [10] that consist of two CNN networks—one generator and one discriminator—have been demonstrated to exhibit better performance on nonlinear intensity transformation between source and target images than traditional nonlinear regression and other neural network approaches [11,12,13]. Research studies have applied GANs models on medical image-to-image translation tasks through learning the mapping between two image distributions. [14,15,16,17,18,19,20,21,22]. The pix2pix GAN [23] was the first success to use a conditional GAN [24] to learn the mapping between paired images. The pGAN [17] employed structural regularization through combining a perceptual loss with pixel-wise loss to enforce a multi-level match between the paired images. In the scenario that multiple paired contrasts are available to synthesize one missing contrast, a multi-stream GAN (mustGAN) [18] was proposed to leverage both shared and complementary features of multiple source images via a mixture of multiple one-to-one streams and a joint many-to-one stream. As paired image sets may be difficult and expensive to acquire, and in some cases impossible, CycleGAN [23] was proposed for unpaired image-to-image translation through a cycle-consistency loss to ensure the forward and backward mapping to be bijections and reverses of each other. CycleGAN has been applied to the synthesis of CT to PET [25,26], MR to CT [19,20], CT to MR [21], and T1w to T2w MRI images [17,22]. More specifically, Wolterink et al. [19] showed CycleGAN trained with unpaired data was able to outperform a GAN model trained with paired brain MR and CT images. Hiasa et al. [20] investigated CycleGAN performance on the number of training data and on the incorporation of consistency loss. Chartsias et al. [21] leveraged synthesized cardiac MR images to achieve improved accuracy in segmentation. Oh et al. [22] used an optimal-transport-driven cycle-consistent generative adversarial network on an unpaired human knee MRI dataset. Dar et al. [17] investigated synthesis performance via pixel-wise perceptual losses and cycle-consistency loss for both registered and unregistered images. It has been demonstrated that the CycleGAN is able to learn an optimal transport between two probabilistic distributions that simultaneously minimizes the statistical distances between the empirical data and synthesized data in two domains.

A novel switchable CycleGAN architecture was developed by designing a single switchable generator using the adaptive instance normalization (AdaIN) technique [27,28]. AdaIN was first proposed to enable arbitrary image style transfer in real-time by aligning the mean and variance of “content” features in images to those of the targeted “style” features [29,30]. The switchable CycleGAN utilized the AdaIN as a switch to control the generator for synthesizing images with different styles. This pioneering design enables a single image generator in the model, compared to two separate image generators in the original CycleGAN model.

One notable limitation of the CycleGAN is that it requires two separate image generators at the training phase to enforce cycle-consistency loss when conducting forward and backward image synthesis. The two separate generators demand more training parameters and time. This constitutes the main hurdle for robust model training. Switchable CycleGAN has been successfully applied in synthesizing CT images in different doses and kernels with less model training time and more stable model performance, even with small samples of unpaired training data [27,28]. However, it remains unclear if switchable CycleGAN can be applied to a task of cross-contrast MRI image synthesis. In addition, whether switchable CycleGAN is able to outperform original CycleGAN on such a task is still an open question.

In this study, we developed a switchable CycleGAN model [27,28] for image synthesis between T1w and T2w brain MRI images. We collected and processed a large set of structural MRI images from a publicly accessible study database [31] as our testbed. Then, we conducted extensive experiments to compare switchable CycleGAN with the original CycleGAN. Both models are trained with unpaired images and are evaluated based on both visual assessments and quantitative metrics (i.e., image synthesis quality, robustness on small datasets, and time efficiency).

## 2. Materials and Methods

### 2.1. MRI Data

We used the publicly available Adolescent Brain Cognitive Development (ABCD) Study database [31] for model development and validation. 1517 subjects with both T1w and T2w MRI scans available were included in the study. Prospective motion correction was originally included in the ABCD image protocol for all structural MRI acquisitions. Both T1w and T2w were acquired using three different 3T MRI scanner manufacturers with the following acquisition parameters: Siemens Healthineers (Prisma VE11B-C): axial T1w images: TR =2500 ms, TE =2.88 ms, flip angle $=8^{{}^{\circ}},$ volume size =256×256×176, voxel dimensions =1.0 mm×1.0 mm×1.0 mm; axial T2w images: TR =3200 ms, TE =565 ms, flip angle = variable, volume size =256×256×176, and voxel dimensions =1.0 mm×1.0 mm×1.0 mm. Philips Healthcare (Achieva, dStream, or Ingenia): axial T1w images: TR =6.31 ms, TE =2.9 ms, flip angle $=8^{{}^{\circ}},$ volume size =256×256×225 , voxel dimensions =1.0 mm×1.0 mm×1.0 mm; axial T2w images: TR =2500 ms, TE =251.6 ms, flip angle $=8^{{}^{\circ}}$, volume sizes =256×256×256, and voxel dimensions =1.0 mm×1.0 mm×1.0 mm. GE (Discovery MR750w, DV25–26): axial T1w images: TR =2500 ms, TE =2 ms, flip angle $=8^{{}^{\circ}},$ volume size =256×256×208 , voxel dimensions =1.0 mm×1.0 mm×1.0 mm; axial T2w images: TR =3200 ms, TE =60 ms, flip angle = variable, volume sizes =256×256×208, and voxel dimensions =1.0 mm×1.0 mm×1.0 mm.

### 2.2. Overview of Switchable CycleGAN

**Figure 1:**
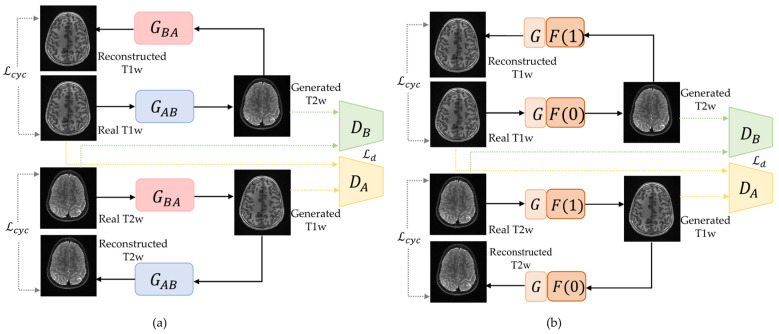
Overview of original CycleGAN and switchable CycleGAN for T1-weighted (T1w) and T2-weighted (T2w) pediatric brain MRI images synthesis. (**a**) The schema of CycleGAN [11] with two different generators $G_{BA}$ and $G_{AB}$. $D_{A}$ is the discriminator that differentiates generated T1w images and real T1w images, and $D_{B}$ is the discriminator that differentiates synthesized T2w images from real T2w images. $L_{cyc}$ is the cycle-consistency loss, and $L_{d}$ is the discriminator loss. (**b**) The schema of switchable CycleGAN with one single generator consists of an image Autoencoder G followed by AdaIN coder F. Discriminators of switchable CycleGAN are the same as CycleGAN.

Suppose that the domain A is composed of T1w brain images, while the images in domain B are T2w brain images. As shown in Figure 1a, a CycleGAN [11] framework for T1w and T2w image synthesis includes two separate generators: one forward generator from T1w images to T2w images ($G_{AB}$), and one backward generator from T2w images to T1w images ($G_{BA}$). In contrast, the switchable CycleGAN designed a single switchable generator for image synthesis between T1w and T2w MRI images. As shown in Figure 1b, the switchable generator includes two modules: Autoencoder G and AdaIN coder F. The Autoencoder module works as a baseline network to achieve the image “content” synthesis between domain A and domain B, while AdaIN coder adjusts the “style” of images as a switch (e.g., F(0) for synthesis from T1w to T2w, and F(1) for synthesis from T2w to T1w).

The premise of AdaIN is that image representation estimation is possible by modifying the mean and variance of the feature map. To be more specific, AdaIN-based image synthesis was performed by matching the mean and variance of the feature map of the input image to those of the reference target image. Given an input feature map is represented by $X=\left[x_{1}\cdots x_{N}\right]\in {\mathbb{R}}^{N\times H\times W},$ where N is the number of channels in the input feature map $x_{n}$, and $x_{n}\in {\mathbb{R}}^{\mathrm{HW}\times 1}$ refers to the n-th column vector of X, which represents the input feature map of size of H×W at the n-th channel. Suppose the feature map of reference target image is represented by Y $=\left[y_{1}\cdots y_{N}\right]\in {\mathbb{R}}^{N\times H\times W}$. After encoding the input images and targeted target images in feature space, an AdaIN layer aligns the mean and variance of $x_{n}$ to match those of $y_{n}$ using the following transformation:

(1) $$z_{n}=T\left(x_{n},y_{n}\right)\;:=\frac{\sigma \left(y_{n}\right)}{\sigma \left(x_{n}\right)}\left(x-\mu \left(x_{n}\right)1\right)+\mu \left(y_{n}\right)1,\;\;n=1,\;\cdots ,\;N$$

where $1\in {\mathbb{R}}^{\mathrm{HW}}$ is the H×W-dimensional vector composed of 1, and $\mu \left(x_{n}\right)$, $\mu \left(y_{n}\right),\;\sigma \left(x_{n}\right)$*,* and $\;\sigma \left(y_{n}\right)$ are the mean and standard deviation, computed across spatial dimensions.

The AdaIN for switching between domain A and domain B  is similar to [27] and can be represented as follows:

(2) $$\left(\mu \left(y\right),\;\sigma \left(y\right)\right)=\{\begin{array}{c} \left(1,0\right),\;\;\;\;\;\;\;\;\;\;\;\;domain\;A\;\\ \left(\mu_{B},\sigma_{B}\right),\;\;\;\;\;\;\;\;domain\;B\;\; \end{array}$$

With Equation (2), AdaIN coder in Figure 1b is defined as:

(3) $$F\left(\gamma \right):=\left[\begin{array}{c} \sigma \left(\gamma \right)\\ \mu \left(\gamma \right) \end{array}\right]=\left(1-\gamma \right)\left[\begin{array}{c} 1\\ 0 \end{array}\right]+\gamma \left[\begin{array}{c} \sigma_{B}\\ \mu_{B} \end{array}\right],\;$$

where $\sigma_{B}$ and $\mu_{B}$ are learnable parameters during network training and γ is a variable that represents the domain. In Equation (2), γ=0 when it represents domain A, and γ=1 when it represents domain B. Then, the synthesis from domain A to domain B can be written as:

(4) $$G_{AB}\left(x\right)=G_{1,0}\left(x\right):=G\left(x;F\left(0\right)\right).$$

The synthesis from domain B to domain A can be described as follows:

(5) $$G_{BA}\left(y\right)=G_{0,1}\left(y\right):=G\left(y;F\left(1\right)\right).$$

### 2.3. Network Architecture of Switchable CycleGAN

#### 2.3.1. Generator

##### Autoencoder Module

**Figure 2:**
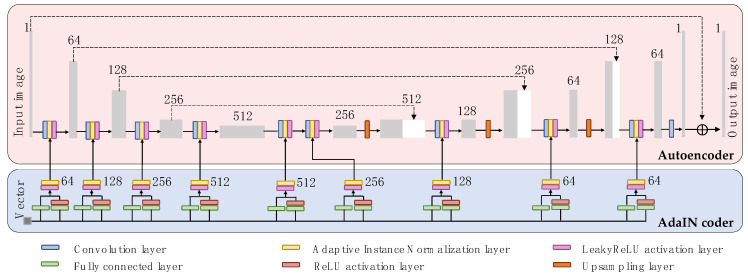
Overview of generator network architecture in switchable CycleGAN. The **upper** part (light red color) is the autoencoder module in the generator. The **lower** part (light blue color) is the AdaIN coder module in the generator.

As shown in Figure 2, the Autoencoder module (light red color), which is based on the U-net architecture [32], consists of a contracting path and an expansive path. The contracting path consists of the repeated applications of four convolution layers for image learning and down-sampling. The four convolution layers are of kernel size 4, stride size 2, and padding size 1. At each down-sampling step, we doubled the number of feature channels. The AdaIN layers take a mean vector and a variance vector as input. Each of four AdaIN layers is followed by a Leaky Rectified Linear Unit (LeakyReLU) layer.

Every step in the expansive path consists of an up-sampling of the feature map followed by a four convolutional layer that halves the number of feature channels. The four convolutional layers are for image reconstructing and up-sampling, and of kernel size 4, stride size 2, and padding size 1. These are followed by a concatenation with the correspondingly cropped feature map from the contracting path, and three 4×4 convolutional layers, each connected with an AdaIN layer and a LeakyReLU layer. The cropping is necessary due to the loss of border pixels in every convolution. At the final layer, a 1 × 1 convolution is used to map each 64-component feature vector to the channel size one.

##### AdaIN Coder Module

As shown in Figure 2, the AdaIN coder module (light blue color) connects to both the encoder and decoder of the Autoencoder module. The AdaIN coder takes a vector of 1×128 size as input and outputs nine pairs of mean and variance vectors. The AdaIN coder consists of two fully connected layers, one Rectified Linear Unit (RELU) layer to prevent the variance vectors from becoming negative, an AdaIN layer, and a LeakyReLU layer. Accordingly, the AdaIN coder is very light-weight. Since the switchable CycleGAN employs a single generator, the number of the model parameters is largely reduced.

#### 2.3.2. Discriminator

**Figure 3:**
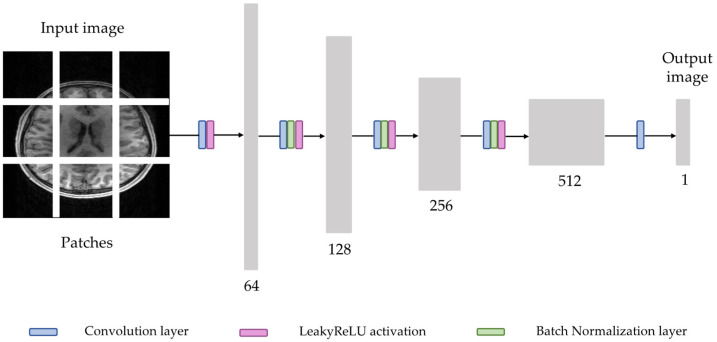
Overview of discriminator network architecture in switchable CycleGAN.

For the discriminator, PatchGAN [23] structure was utilized to classify whether overlapping image patches are real or generated (Figure 3). Such patch-level discriminator architecture not only has fewer parameters than a full-image discriminator but also can work on arbitrarily sized images [33]. The discriminator consists of five convolution layers, in which the first convolution layer uses a stride of 2, while the following four convolution layer use a stride of 1. The first convolution layer is followed by the LeakyReLU layer, and other convolution layers are followed by batch normalization layers and LeakyReLU layers, except for the last convolution layer. The first convolution layer gets an input image with one channel and generates a feature map with 64 channels. After that, each time the feature map passes through the convolution layer, the number of channels is doubled. In the last layer, the output tensor is obtained by reducing the number of channels to size one.

### 2.4. Model Training

The switchable CycleGAN model for T1w and T2w image synthesis was trained in a similar manner to CycleGAN network [11]. We trained the model by solving the following min-max optimization problem [10]:

(6) $$G^{*},F^{*}=\arg\underset{G,F}{\;\min}\underset{D_{A},D_{B}}{\max}L_{total}\left(G,F,D_{A},D_{B}\right).$$

The total loss objective is:

(7) $$L_{total}\left(G,F,D_{A},D_{B}\right)=\lambda_{adv}L_{adv}\left(G,F,D_{A},D_{B}\right)\;+\lambda_{cyc}L_{cyc}\left(G,F\right)\;+\lambda_{id}L_{id}\left(G,F\right),$$

where $\lambda_{adv}$ is the weight parameter of adversarial loss, $\lambda_{cyc}$ is the weight parameter of cycle-consistency loss, and $\lambda_{id}$ is the weight parameter of identity loss. For $L_{adv}$ in Equation (7), we used least-square loss [34] same as CycleGAN. This least-square loss was more stable during training and generated higher quality results [11]. The adversarial loss is represented as follows:

(8) $$L_{adv}\left(G,F,D_{A},D_{B}\right)\;=E_{y\sim P_{A}}\left[||D_{A}{\left(y)|\right|}_{2}^{2}\right]+E_{x\sim P_{B}}\left[||1-D_{A}{\left(G_{1,0}\left(x\right))|\right|}_{2}^{2}\right]\;=E_{x\sim P_{B}}\left[||D_{B}{\left(x)|\right|}_{2}^{2}\right]+E_{y\sim P_{A}}\left[||1-D_{B}{\left(G_{0,1}\left(y\right))|\right|}_{2}^{2}\right],$$

where ${\left||\cdot |\right|}_{2}$ is the $l_{2}\;norm;\;G_{1,0}\left(x\right)$ and $G_{0,1}\left(y\right)$ are defined in Equation (4) and Equation (5). $D_{A}$ is the discriminator differentiates generated T1w images and real T1w images, and $D_{B}$ is the discriminator differentiates synthesized T2w images with real T2w images. The cycle-consistency loss is defined as

(9) $$L_{cyc}\left(G,F\right)=E_{y\sim P_{A}}\left[||G_{1,0}\left(G_{0,1}\left(y\right)\right)-{y||}_{1}\right]\;+E_{x\sim P_{B}}\left[||G_{0,1}\left(G_{1,0}\left(x\right)\right)-{x||}_{1}\right].$$

We used identity loss to encourage the mapping when real samples of target domain are provided as the input of the generator. The identity mapping is simulated as follows:

(10) $$L_{id}\left(G,F\right)=E_{y\sim P_{B}}\left[||G_{1,0}\left(y\right)-{y||}_{1}\right]\;+E_{x\sim P_{A}}\left[||G_{0,1}\left(x\right)-{x||}_{1}\right].$$

The discriminators were trained to minimize the adversarial loss $L_{adv}\left(G,F,D_{A},D_{B}\right),$ while the generator G is trained to maximize it. The generator and discriminators are updated alternatively for adversarial training.

### 2.5. Implementation Details

For training, we iteratively trained a switchable CycleGAN with 200 epochs. All networks were trained using the optimizer ADAM solver [35] with $\beta_{1}=0.5$, $\beta_{2}=0.999$. The learning rate for the first 100 epochs was 0.0002, and the learning rate linearly decayed to zero over the next 100 epochs. The minibatch size was 1. For the hyperparameters in Equation (7), the loss weights $\lambda_{cyc}$, $\lambda_{id}$, and $\lambda_{adv}$ were set to 10, 5, and 1, respectively. The model was trained on NVIDIA GeForce RTX 3080.

All the methods in this study were implemented in Pytorch v1.9.1. The input images were randomly cropped into small patches of size 128×128 during the training. They were also randomly flipped both horizontally and vertically for data augmentation and model generalizability. Training images were provided in a randomized unpaired way, making it unlikely that both an T1w image and its registered corresponding T2w image were simultaneously shown to GAN model. We also followed Shrivastava et al.’s strategy [36] and updated the discriminators using a history of generated images rather than the ones produced by the latest generators. An image buffer was implemented to store the 50 previously synthesized images.

### 2.6. Model Evaluation and Statistical Analysis

Multi-contrast T1w and T2w MRI images from a given subject were registered using advanced normalization tools [37]. We extracted 10 slices of brain MRI images from each subject, resulting in a total of 30,340 slices. We randomly selected 1063 subjects (70%) for training, 151 subjects (10%) for validation, and 303 subjects (20%) for testing. We compared switchable CycleGAN with baseline CycleGAN [11], as well as pix2pix GAN models [23]. Tuning hyperparameters in deep neural networks, especially in complicated models such as GANs, can be computationally intensive [38,39]. Thus, it is quite common in deep learning research to perform one-fold cross-validation [40,41] or even directly adopt hyperparameter selection from published work [19,25,41]. We adopted hyperparameters of switchable CycleGAN from a prior study [27]. The epoch numbers (in the range [100, 200]) were selected based on performance of the validation set through on-fold cross-validation. Both methods are compared with the same training and test set data.

We used the structural similarity index (*SSIM*) [42] and peak signal-to-noise ratio (*PSNR*), two well-known metrics, to evaluate the quality of synthesized images. The equation for *PSNR* calculation is as follows:

(11) $$PSNR\left(x,\hat{x}\right)=20log_{10}\frac{MAX_{x}}{∥x-\hat{x}{∥}_{2}},$$

where $MAX_{x}$ is the maximum possible value of image x. The *SSIM* is calculated by the equation:

(12) $$SSIM\left(x,\hat{x}\right)=\frac{\left(2\mu_{x}\mu_{\hat{x}}+c_{1}\right)\left(2\sigma_{x\hat{x}}+c_{2}\right)}{\left(\mu_{x}^{2}+\mu_{\hat{x}}^{2}+c_{1}\right)\left(\sigma_{x}^{2}+\sigma_{\hat{x}}^{2}+c_{2}\right)},$$

where μ is mean of the image, σ is variance of the image, and $\sigma_{x\hat{x}}$. is covariance of the images *x* and $\hat{x}.\;L$ is the dynamic range of the pixel intensities, and the two variables are defined by $c_{1}={\left(k_{1}L\right)}^{2},\;c_{2}={\left(k_{2}L\right)}^{2}$, which are used to stabilize the division. We used $k_{1}=0.01,\;k_{2}=0.03$ as in the original work [42].

To compare different image generative models, we conducted nonparametric Wilcoxon signed-rank tests to test the performance difference. A *p*-value less than 0.05 is considered statistically significant. All statistical analyses were conducted in Python 3.8.5 and SciPy 1.7.3.

## 3. Results

### 3.1. Quantitative Comparison between CycleGAN and Switchable CycleGAN

Table 1 presents PSNR and SSIM data across test images synthesized using CycleGAN and switchable CycleGAN. For T1w to T2w image synthesis, the switchable CycleGAN method was 1.2 dB higher (p-value <0.001) in PSNR than CycleGAN. For the image synthesis from T2w to T1w, switchable CycleGAN was 0.1 dB (p-value <0.001) higher in PSNR. The PSNR for switchable CycleGAN in two directions was an average of 0.65 dB higher than CycleGAN. As for SSIM T1w to T2w image synthesis, switchable CycleGAN model was 9.5% higher (p-value <0.001) than CycleGAN, while for the image synthesis from T2w to T1w, switchable CycleGAN was 12.5% higher (p-value <0.001) than CycleGAN. The SSIM for switchable CycleGAN in two directions was, on an average, 11.0% higher than CycleGAN. Considering two synthesis directions together, pix2pix GAN was 0.0023 higher (p-value <0.001) in SSIM and 0.002 dB higher (p-value <0.001) in PSNR than CycleGAN. Switchable CycleGAN outperformed pix2pix GAN, being 0.05 higher in SSIM (p-value <0.001) and 0.652 dB higher in PSNR (p-value <0.001). This demonstrated that switchable CycleGAN quantitatively outperformed CycleGAN in image synthesis of T1w and T2w pediatric brain images. This also demonstrated that switchable CycleGAN trained with unpaired data outperformed pix2pix GAN trained with paired data. Since the main hypothesis of this work is to investigate difference between CycleGAN and switchable CycleGAN using unpaired data, henceforth we will only focus on experiments with models using unpaired data.

### 3.2. Visualization

**Figure 4:**
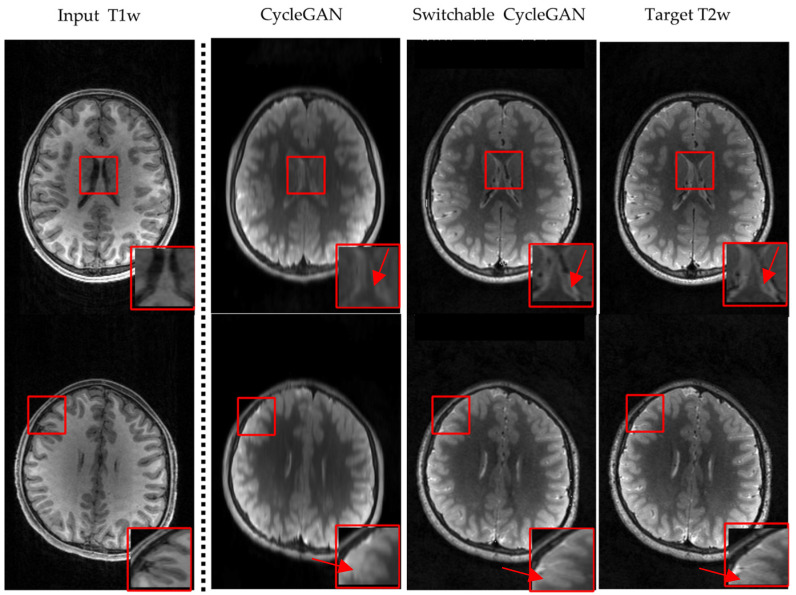
Visual comparison results by CycleGAN and switchable CycleGAN on the ABCD Study Dataset, T1w to T2w image synthesis. Different rows display two individual brain MRI images.

**Figure 5:**
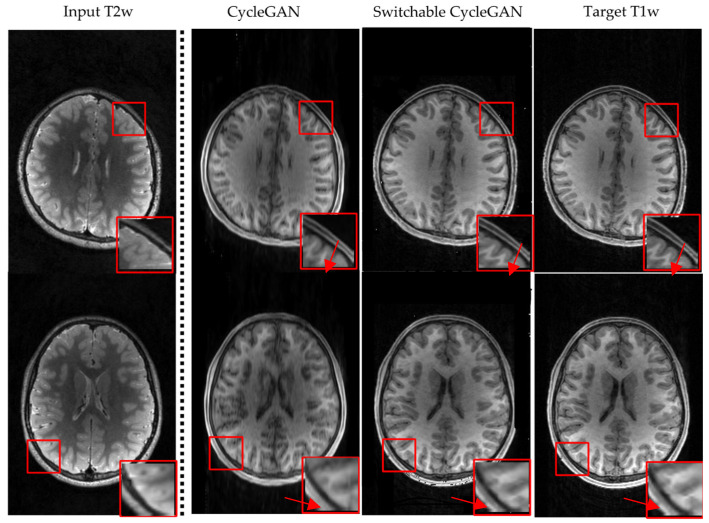
Visual comparison results by CycleGAN and switchable CycleGAN on the ABCD Study Dataset, T2w to T1w image synthesis. Different rows display two individual brain MRI images.

We compare visualization results in Figure 4 and Figure 5. Figure 4 is the T1w to T2w image synthesis, and Figure 5 is the perspective of T2w to T1w image synthesis. For both directions, the results generated by switchable CycleGAN are more consistent with the target images and could remain sophisticated structures and preserve more details of brain tissues than CycleGAN as the red arrows point to. In particular, in the red box of comparison results, we observed that the images generated by CycleGAN have some artifacts and missing details. These results demonstrate that switchable CycleGAN is also superior qualitatively to CycleGAN in synthesizing T1w and T2w images.

### 3.3. Robustness to Small Dataset

Since switchable CycleGAN utilizes a single generator, the number of parameters of the model are reduced, which results in robust training, even for small datasets. We set out to investigate the robustness of two generative models to various training sizes using the ABCD dataset. We varied the number of image samples in the dataset as 300, 3000, and 30,000. We then calculated the SSIM results of CycleGAN and switchable CycleGAN (Table 2).

CycleGAN suffers from greater loss in SSIM performance as compared to switchable CycleGAN as dataset size decreases. From data size 30,000 to 300, the SSIM of CycleGAN dropped 15.2% for the synthesis from T1w to T2w images and decreased 23.4% for the synthesis from T2w to T1w images; comparatively, SSIM values for switchable CycleGAN demonstrated less dramatic decreases of 8.81% and 12.4%, respectively. This illustrates that switchable CycleGAN is more robust on small datasets than CycleGAN. For the t-test on the SSIM results between CycleGAN and switchable CycleGAN, the p-values are less than 0.001 when the number of image samples in the dataset are 300, 3000, and 30,000. From these analyses, we could see that switchable CycleGAN shows significantly improved performance in generating T1w and T2w MR brain images.

### 3.4. Time Efficiency

We further investigated the training time efficiency of switchable CycleGAN. We timed the training process of 30,000 dataset size on one single NVIDIA GeForce RTX 3080 GPU, and the training epochs in the two methods were both 200 epochs.

Table 3 shows that the training time of switchable CycleGAN is 50.3% less than CycleGAN under the same experiment settings, indicating that switchable CycleGAN outperforms CycleGAN in model training efficiency.

## 4. Discussion

To develop a deep learning model that performs cross-contrast MRI image synthesis, it is desirable to collect a large dataset of paired training data to train a generative model (e.g., GAN) [12,17]. However, collecting all paired MRI images for different scanners, imaging protocols, and conditions is a very challenging task that requires careful data collection plans. Thus, we are particularly interested in developing image synthesis models that utilize unpaired data. CycleGAN has achieved promising results on a number of image synthesis tasks without paired data [17,19,20,21,22]. More recently, a novel switchable CycleGAN was developed to reduce the model complexity of CycleGAN so as to improve the model training efficiency, and its effectiveness has been demonstrated using CT data [27,28]. Here, we conducted a comprehensive evaluation of the switchable CycleGAN using a large dataset of T1w and T2w images.

We believe this is the first study to develop a switchable CycleGAN model for multi-contrast T1w-T2w structural MRI synthesis. The main innovation of switchable CycleGAN is that it designs an AdaIN coder (Figure 2) outside the autoencoder module (Figure 2). The benefit of this design is twofold. First, it reduces the number of generators from two to one. Consequently, this decreases the trainable parameters and computational time. Second, it improves image quality and model robustness on smaller datasets due to decreased model complexity. Previously, these benefits have been illustrated with CT data [27,28]. In the current work, our results seem consistent with prior findings. We observed that switchable CycleGAN outperformed the original CycleGAN model with regards to image synthesis quality, robustness on small datasets, and time efficiency.

It is beneficial to design a U-net as the Autoencoder module within the switchable generator (Figure 2). In this way, MRI image features from the contracting path layers are combined with expansive path layers. The input image features can be easily taken into account by the generator so that the brain structure (“image content”) of the real MRI images can be attained by the generated MRI images [43]. This attribute is appealing for our image-to-image task: we expect our model to maintain the same structure of brain tissue. In addition, the skip-connections in U-net can mitigate the gradient vanishing/exploding problem, which often haunts deep learning models [44].

Similar to [27,28], in this paper, we used both identity and cycle-consistency loss. The identity loss, which is equal to the autoencoder loss, plays a role in preserving the structure with target domain images by providing pixel-wise constraints. The cycle-consistency loss also poses a strong pixel-wise constraint in that it forces self-consistency when reverting to the original domain, which prevents unexpected brain structures from being created. The generative models (e.g., GANs) lack these two constraints, and it has been reported that falsified structures were observed [27,28]. Thus, we believe that both identity and cycle-consistency loss have their own contributions during model training.

In our experiments, we observed that switchable CycleGAN outperformed baseline CycleGAN in terms of PSNR, SSIM, robustness on small datasets, and time efficiency. We believe that the image quality improvement is mainly due to the inclusion of AdaIN layers. AdaIN [29] was first proposed to better control image style transfer by adjusting the mean and variance of images [30]. Despite its simplicity, AdaIN has been formally justified by recent theoretical work [45] in which image to image translation by AdaIN implements the optimal transport map between two spatial distributions of image features, which are equipped with the i.i.d. Gaussian distributions. Therefore, AdaIN finds the optimal approximations of transport map from the input image distribution to the reference target image distribution. The model efficiency improvement is mainly attributed to the design of switchable AdaIN-enabled single shared generator. The shared generator enables the common latent representation learning of two contrasts and boosts the cross-contrast correlation learning. Compared to two generators in CycleGAN, the single shared image generator of switchable CycleGAN leads to a tremendous reduction in the trainable network parameters, which accelerates the training process and, in turn, enables handling of overfitting issues with relatively smaller training datasets. Such desirable robustness and reliability make the switchable CycleGAN a more practical solution for multi-contrast T1w and T2w structural MRI synthesis.

It is also noticed that the performance of pix2pix GAN heavily relies on the quality of image registration. Unfortunately, there is typically a lack of perfect medical image registration approaches. Any less-than-perfect registered image pairs may influence the performance of pix2pix GAN. Our proposed unpaired switchable CycleGAN outperformed paired pix2pix GAN. Besides the contributions of AdaIN laters, such performacne improvement also partially attributes to the CycleGAN’s cycle-consistent loss, which facilitates learning the mapping between two contrasts without paired data supervision. This mitigates the impact of less-than-perfect registered image pairs.

The multi-contrast data have been registered prior to modeling efforts. We do not expect this registration step to influence training in CycleGAN and switchable CycleGAN as training images were provided in a randomized unpaired way, making it unlikely that both a T1w/T2w image and its registered corresponding T1w/T2w image were simultaneously shown to the GAN model. In addition, images were randomly cropped into small patches of size 128×128  and randomly flipped both horizontally and vertically during the training, which partially cancels the efforts of registration. The registration is mostly and mainly for the test set, to make the testing evaluation metrics values more accurate and trustworthy. The same training strategy can be found in [11,19].

Our study has some limitations. First, there is large data heterogeneity in our testbed multi-contrast MRI data. As shown in Section 2, data were collected from the largest pediatric brain study, and their MRI data were acquired using multiple scanners from three different vendors. The scanner bias might be a confounding factor that impacts the quality of generated images. However, we believe this presented a good opportunity to test the generalizability of switchable CycleGAN without using well-planned, well-harmonized training data. Second, although generated MRI images using switchable CycleGAN demonstrated higher SSIM and PSNR values than the original CycleGAN model, much work remains in the area of cross-contrast image synthesis. The highest SSIM value generated by switchable CycleGAN was 0.7468. Further investigations can be conducted to improve the quality of generated MRI images. Third, it is unclear how the model synthesizes brain pathology, if there is any, in the brain MRI images. This is an interesting study that requires a large scale of MRI images with pathological regions. Finally, we only focused on a portion of brain tissues (10 slices of axial brain MRI images in each subject). Further study may be necessary to synthesize the whole volume of the pediatric brain. In the current study, we mainly focused on providing a unified environment to conduct a fair comparison between switchable CycleGAN and original CycleGAN.

## 5. Conclusions

In this paper, we conducted pediatric brain image synthesis between T1w and T2w MRI data, which, to our best knowledge, is the first multi-contrast MRI image synthesis study using switchable CycleGAN model. The model performance was evaluated both quantitively and qualitatively. Experimental results demonstrate that switchable CycleGAN outperformed the original CycleGAN and pix2pix GAN models with higher PSNR and SSIM. We further illustrated that switchable CycleGAN was more robust on small datasets than CycleGAN model. Additional time efficiency analysis showed that training time of switchable CycleGAN was 50.3% less than that of CycleGAN.

The proposed work can be extended to generate super-resolution MRI images, as in [30], where AdaIN was used to modify the relative importance of features for the subsequent convolution operation to synthesize higher spatial resolution (e.g., 512×512, 1024×1024). The proposed work can also be implemented for three-modality learning, as in [27]. As the AdaIN is able to disentangle arbitrary high-level attributes in source and target modalities, the image synthesis between T1w and T2w can be naturally extended into the conversion among T1w, T2w, and diffusion-weighted imaging, as well as other non-standard contrasts. The performance of the proposed switchable CycleGAN may be further enhanced by incorporating transformer blocks [46,47] as transformers are proven to be robust in natural language processing and computer vision domains.

## Figures and Tables

**Table 1:** Quantitative evaluations of peak signal-to-noise ratio (PSNR) and structural similarity index (SSIM) for CycleGAN and switchable CycleGAN for pediatric T1w and T2w images. Higher values indicate better performance.

Method	PSNR		SSIM	
	T1w→T2w	T2w→T1w	T1w→T2w	T2w→T1w
CycleGAN [11]	30.481 ± 1.296	31.614 ± 0.620	0.682 ± 0.150	0.651 ± 0.113
pix2pix GAN [23]	31.373 ± 1.443	30.726 ± 0.544	0.691 ± 0.131	0.688 ± 0.120
Switchable CycleGAN	31.671 ± 1.813	31.733 ± 1.093	0.747 ± 0.169	0.732 ± 0.128

**Table 2:** Structural similarity index (SSIM) evaluation of different data sizes using CycleGAN versus switchable CycleGAN. For SSIM, higher values indicate better performance.

Data Size	CycleGAN [11]		Switchable CycleGAN	
	T1w→T2w	T2w→T1w	T1w→T2w	T2w→T1w
30,000	0.682 ± 0.150	0.651 ± 0.113	0.747 ± 0.169	0.732 ± 0.128
3000	0.614 ± 0.214	0.587 ± 0.146	0.696 ± 0.156	0.686 ± 0.130
300	0.578 ± 0.126	0.498 ± 0.158	0.681 ± 0.136	0.641 ± 0.135

**Table 3:** Training time efficiency comparison between CycleGAN and Switchable CycleGAN.

Method	Training Time (Number of Hour of 200 Epochs)
CycleGAN [11]	74.4
Switchable CycleGAN	36.9

## Data Availability

The data in this study are publicly available brain MRI images from the ABCD study.
